# Bioinformatics Analysis of Common Differential Genes of Viral Myocarditis and Dilated Cardiomyopathy: Screening for Potential Pharmacological Compounds

**DOI:** 10.3390/jcdd9100353

**Published:** 2022-10-15

**Authors:** Junyi Zhang, Mingzhu Xu, Tan Chen, Yafeng Zhou

**Affiliations:** Department of Cardiology, Dushu Lake Hospital Affiliated to Soochow University, Suzhou 215000, China

**Keywords:** viral myocarditis, dilated cardiomyopathy, precision medicine, bioinformatics analysis

## Abstract

(1) Background: The mechanism of viral myocarditis (VMC) progression to dilated cardiomyopathy (DCM) remains unclear. The aim of this study was to identify key genes in the progression of VMC to DCM, so as to find potential therapeutic drugs and provide insights for future research. (2) Methods: Differential expression analysis of GSE4172 and GSE17800 from the Gene Expression Omnibus (GEO) database was performed using GEO2R, which contained genome-wide analysis of myocardial biopsies from VMC and DCM, respectively. We used the Venn diagram analysis to screen the common differentially expressed genes (DEGs). GO functional enrichment analysis and KEGG pathway analysis were also performed. Then we conducted protein–protein interaction (PPI) networks using STRING and identified hub genes using Cytoscape. Finally, we used cMAP to screen out candidate compounds targeting these hub genes; (3) Results: In total, 2143 DEGs for VMC and 1365 DEGs for DCM were found. Then a total of 191 common DEGs were identified. Biological processes and pathway involved in these genes mainly include GABA−gated chloride ion channel activity and Rap1 signaling pathway. A total of 14 hub genes were identified. PPI network showed these hubs mainly enriched in regulation of WNT signaling pathway and GABA-gated chloride ion channel activity. Subgroup analysis of Severe VMC cohort revealed 10 hub genes which mainly clustered in GABA channel activity, extracellular matrix remodeling and sarcomere dysfunction. Using cMAP, we obtained top 10 potential medications, but only amlodipine is currently viable; (4) Conclusions: Our study finds the hub genes and reveals the important role of GABA-gated chloride ion channel, Rap1 signaling pathway, WNT signaling pathway, extracellular matrix remodeling and sarcomere dysfunction in the progression from VMC to DCM. Amlodipine is a potential viable drug in preventing the progression of VMC to DCM.

## 1. Introduction

Viral myocarditis (VMC) is a localized or diffuse inflammatory disease of the myocardium associated with viral infection, which may have an acute or chronic course [1]. Most patients have a good prognosis, but 10%–15% may progress to dilated cardiomyopathy (DCM) [2]. DCM is a myocardial disease characterized by unilateral or bilateral enlargement of heart cavities and decreased myocardial contractility. Its clinical manifestations are mainly progressive congestive heart failure, often accompanied by various types of arrhythmias, which has a serious impact on patients’ quality of life and life expectancy [3]. Currently, there is a lack of effective clinical treatment. Patients with dilated cardiomyopathy have only a 55% 5-year survival rate with current heart failure therapy, suggesting an urgent need for targeted therapy strategies [4].

The viruses known to cause myocarditis include coxsackie virus (A,B), echovirus, adenovirus, influenza virus (A,B), cytomegalovirus, and chiropoliovirus [5]. The hypothesis of the progression from VMC to DCM has been proposed for a long time. Kawai postmortem examined 44 DCM patients, 8 of whom had definite histological myocarditis [6]. It was found that neutralizing antibody titers of Coxsackie GROUP B (CB) virus were greater than 1:1024 in approximately 30% of DCM patients [7]. Of 165 serologically confirmed CB viral myocarditis patients with long-term follow-up, 13% developed DCM [8]. Due to the close relationship between VMC and DCM, it is often impossible to separate them completely in clinical practice, so some scholars believe that they are different stages of the same pathological process. However, the actual pathogenesis is not well understood. In recent years, with the great improvement of molecular biology techniques such as nucleic acid hybridization, polymerase chain reaction (PCR), endocardial biopsy, and modern immunohistochemistry, more evidence has been obtained on the mechanism of progression from VMC to DCM. Virus infection, immune response, and apoptosis are considered to be important factors [9,10,11]. However, the key genes in the pathogenesis from VMC to DCM have not been identified so far.

In this study, through bioinformatics, we searched for the key genes in the transition from viral myocarditis to non-familial dilated cardiomyopathy by screening the common differentially expressed genes (DEGs) between VMC and DCM, with the hope of finding potential drugs that could prevent the progression of VMC to DCM to some extent and providing clues for further research.

## 2. Materials and Methods

### 2.1. Acquisition of Data of Gene Expression Profiles

The datasets of gene expression profiles GSE4172 and GSE17800 were acquired by logging in the Gene Expression Omnibus (GEO) database (accessed on 5 June 2022, https://www.ncbi.nlm.nih.gov/gds/) [12,13]. In GSE4172, there were 8 samples from VMC patients infected with parvovirus B19 and 4 samples from controls. In GSE17800, the number of samples from DCM patients and controls was 40 and 8, respectively. Both of the two chips were from endomyocardial biopsies (EMBs), the detailed information of which is shown in Table 1.

### 2.2. Processing of Raw Data and Screening of Differentially Expressed Genes (DEGs)

We used fold change and corrected *p*-values to draw Volcano plots. Boxplots were drawn using the R package ggplot2; Expression heatmaps were drawn using the R package heatmap. DEGs were analyzed using the GEO2R tool. We defined *p* < 0.05 and |log2 fold change (FC)| > 0.58 (namely fold change >1.5 times) as statistically significant DEGs.

### 2.3. Screening of Common Differentially Expressed Genes

The differential genes selected from the two chips underwent Venn diagram analysis and a minority of DEGs shared by both were defined as common DEGs, which will be used for subsequent analysis.

### 2.4. Gene Ontology (GO) Enrichment Analysis and Kyoto Encyclopedia of Genes and Genomes (KEGG) Pathway Analysis

GO functional enrichment analysis which included molecular function (MF), biological process (BP), and cellular component [CC] analysis and KEGG pathway analysis of the DEGs were performed using the R package clusterProfiler.

### 2.5. Construction of Protein–Protein Interaction (PPI) Networks and Screening of Hub Genes

PPI network analysis was performed for the common DEGs of VMC and DCM by using String (accessed on 6 June 2022, https://www.string-db.org/) to predict the interaction between proteins encoded by DEGs. Confidence interaction score was set at 0.15 for the significant criterion. According to the result of PPI network, we used Cytoscape and the MCODE module to screen the hub genes.

### 2.6. Screening of Potential Pharmacological Targets

The ConnectivityMap (cMAP) (accessed on 6 June 2022, https://clue.io) database contains data on changes in gene expression profiles caused by more than 30,000 small-molecule compounds acting on multiple cell lines and can be used to compare similarities between drug-induced gene profiles and gene expressions, with connectivity scores ranging from −100 to 100. A positive score suggests that compounds can cause changes similar to those in uploaded genes; a negative score suggests that the compound causes changes opposite to those in the uploaded genes. Therefore, for those up-regulated genes, a negative score suggests that the compound may have a therapeutic effect on the disease. On the contrary, for those down-regulated genes, a positive score suggests that the agent may have a therapeutic effect on the disease. Agents with absolute value of the connectivity scores >80 were considered promising predictors.

### 2.7. Subgroup Analysis

We additionally selected patients with severe myocarditis (sVMC) from GSE4172 for subgroup analysis. We defined sVMC as an EF < 40%. Four patients met the definition of sVMC.

## 3. Results

### 3.1. Screening of Differentially Expressed Genes

#### 3.1.1. Screening of Differentially Expressed Genes in GSE417

**Figure 1:**
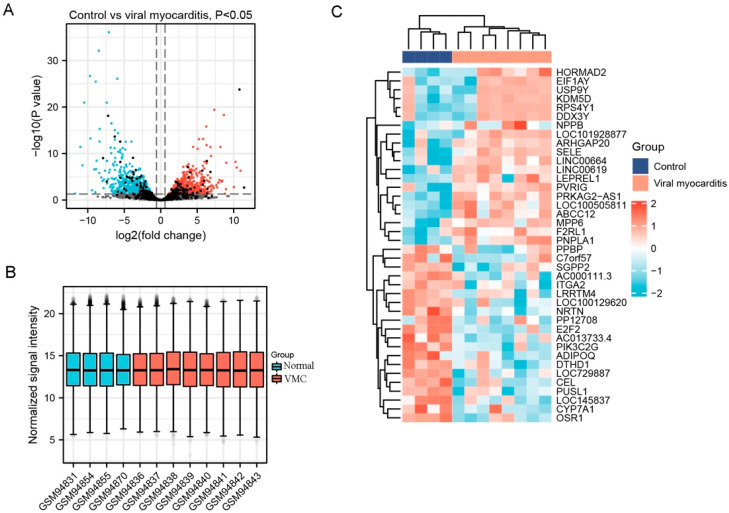
Differentially expressed genes (DEG) analysis on viral myocarditis (VMC). (**A**) Volcanic plots of gene expression of VMC in GSE4172 using ggplot2 package of R 3.6.3. Red represents upregulated DEGs, blue represents downregulated DEGs, grey represents genes which are not differentially expressed. (**B**) Cross comparability evaluation of microarray data using ggplot2 package of R 3.6.3. (**C**) Heat map of the former 50 DEGs using ComplexHeatmap package of R 3.6.3.

A total of 2143 DEGs were selected from GSE4172, including 1217 up-regulated genes and 926 downregulated genes. A part of the major DEGs is presented in Table 2. Clustering analysis of these DEGs was performed, as shown in the volcano plot (Figure 1A). Data normalization and cross-comparability were also evaluated. As shown in Figure 1B, the selected samples were centered and the numerical distribution met the standard, indicating that the microarray data were of high quality and had cross-comparability. Based on the *p*-value, the former 50 DEGs with the lowest *p*-values were analyzed in the heatmap (Figure 1C).

#### 3.1.2. Screening of Differentially Expressed Genes in GSE17800

**Figure 2:**
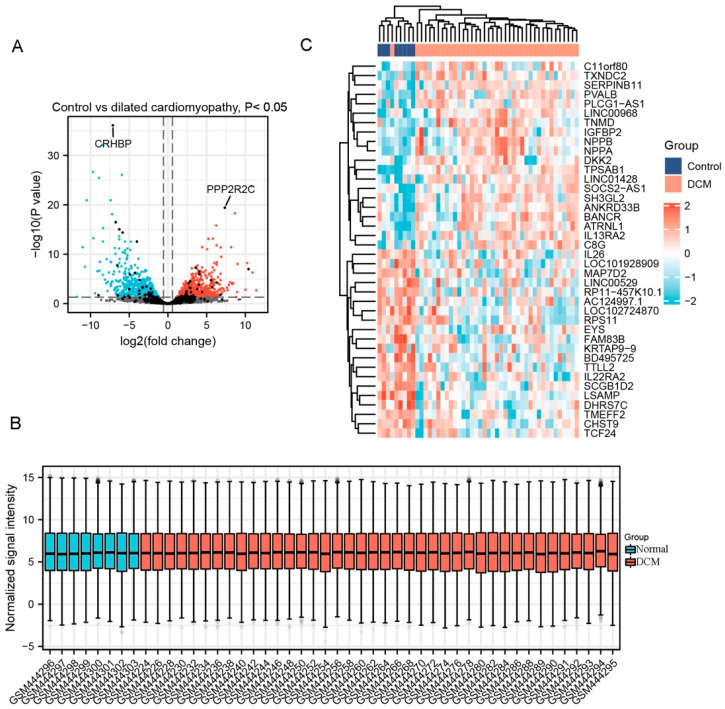
Differentially expressed genes (DEG) analysis on dilated cardiomyopathy (DCM). (**A**) Volcanic plots of gene expression of DCM in GSE17800 using ggplot2 package of R 3.6.3. Red represents upregulated DEGs, blue represents downregulated DEGs, grey represents genes which are not differentially expressed. (**B**) Cross comparability evaluation of microarray data using ggplot2 package of R 3.6.3. (**C**) Heat map of the former 50 DEGs using ComplexHeatmap package of R 3.6.3.

Totally, 1365 DEGs were selected from GSE17800, of which 869 were upregulated genes and 496 were downregulated genes. A part of major DEGs is presented in Table 3. Similarly, clustering analysis, data normalization and cross comparability and heatmap of these DEGs were also performed. As a result, the volcano plot is shown in Figure 2A. The box plot showing a concentrated sample distribution is presented in Figure 2B. The heatmap of the former 50 DEGs with the lowest *p*-values was shown in Figure 2C.

#### 3.1.3. Screening of Common Differentially Expressed Genes

**Figure 3:**
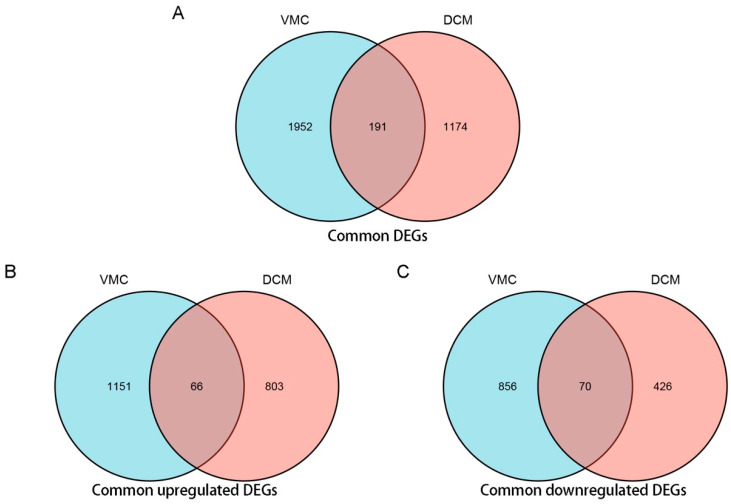
Venn analysis of common differentially expressed genes (DEG) of VMC and DCM using ggplot2 package of R 3.6.3. (**A**) Venn diagram of common DEGs of VMC and DCM. (**B**) Venn diagram of common up-regulated DEGs of VMC and DCM. (**C**) Venn diagram of common down-regulated DEGs of VMC and DCM. (Filter criteria: |log2FC| > 0.58; *p*-Value < 0.05. For upregulated DEGs, the log2FC >0; For downregulated DEGs, the log2FC <0).

Using Venn analysis, a total of 191 common DEGs were screened from GSE4172 and GSE17800 (Figure 3A). Among these genes, 66 were common up-regulated DEGs (Figure 3B) and 70 were common down-regulated DEGs (Figure 3C). The former ten common DEGs with the highest average |log2FC| are shown in Table 4.

### 3.2. GO Enrichment and KEGG Pathway Analysis

**Figure 4:**
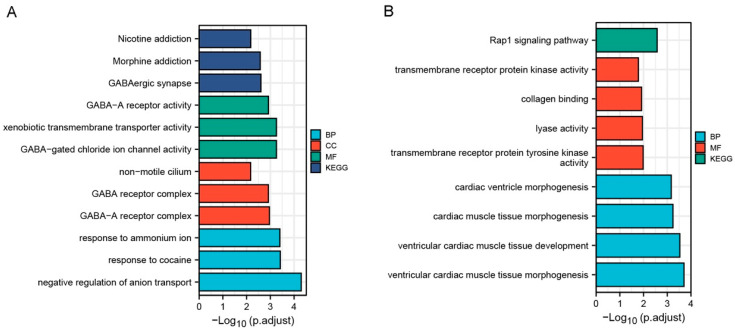
Results of GO enrichment analysis and KEGG pathway analysis for common differentially expressed genes (DEG) of VMC and DCM using clusterProfiler package of R 3.6.3. (**A**) Results of GO enrichment analysis and KEGG pathway analysis for common up-regulated DEGs of VMC and DCM. (**B**) Results of GO enrichment analysis and KEGG pathway analysis for common down-regulated DEGs of VMC and DCM.

The gene ontology enrichment analysis of the common up-regulated DEGs of VMC and DCM were mainly enriched in biological process (BP), cellular localization (CL), and molecular function (MF), especially in “negative regulation of anion transport” (GO-ID:1903792, *p* = 4.99 × 10^−5^), “GABA receptor complex” (GO-ID:1902710, *p* = 0.001), “GABA−gated chloride ion channel activity” (GO-ID:0022851, *p* = 5.51 × 10^−4^) and “xenobiotic transmembrane transporter activity” (GO-ID:0042910, *p* = 5.51 × 10^−4^). KEGG showed that these genes were mainly involved in GABAergic synapse (Table 5, Figure 4A). These results suggest that GABA ion channel transport function and xenobiologic transmembrane transport function are enhanced in patients with VMC and DCM compared with normal controls.

The gene ontology enrichment analysis of the common down-regulated DEGs of VMC and DCM were mainly enriched in biological process (BP) and molecular function (MF), especially in “ventricular cardiac muscle tissue morphogenesis” (GO-ID:0055010, *p* = 1.96 × 10^−4^) and “ventricular cardiac muscle tissue development” (GO-ID:0003229, *p* = 2.94 × 10^−4^). KEGG enrichment results showed that these genes were mainly involved in Rap1 signaling pathway (Table 6, Figure 4B). These results suggest that ventricular myocardial tissue development and Rap1 signaling pathway are inhibited in patients with VMC and DCM.

### 3.3. PPI Network Analysis and Screening of Hub Genes

**Figure 5:**
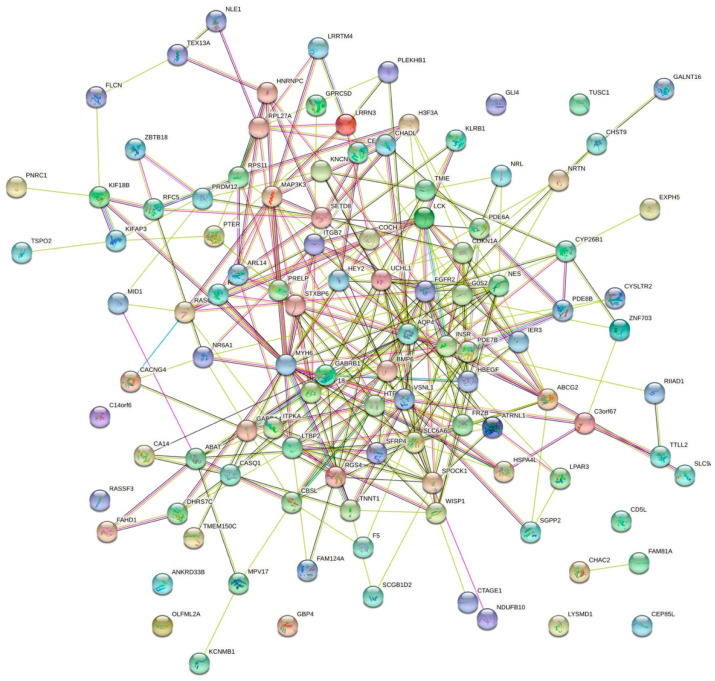
Results of the protein–protein interaction (PPI) network analysis of common differentially expressed genes (DEG) of VMC and DCM constructed using STRING.

**Figure 6:**
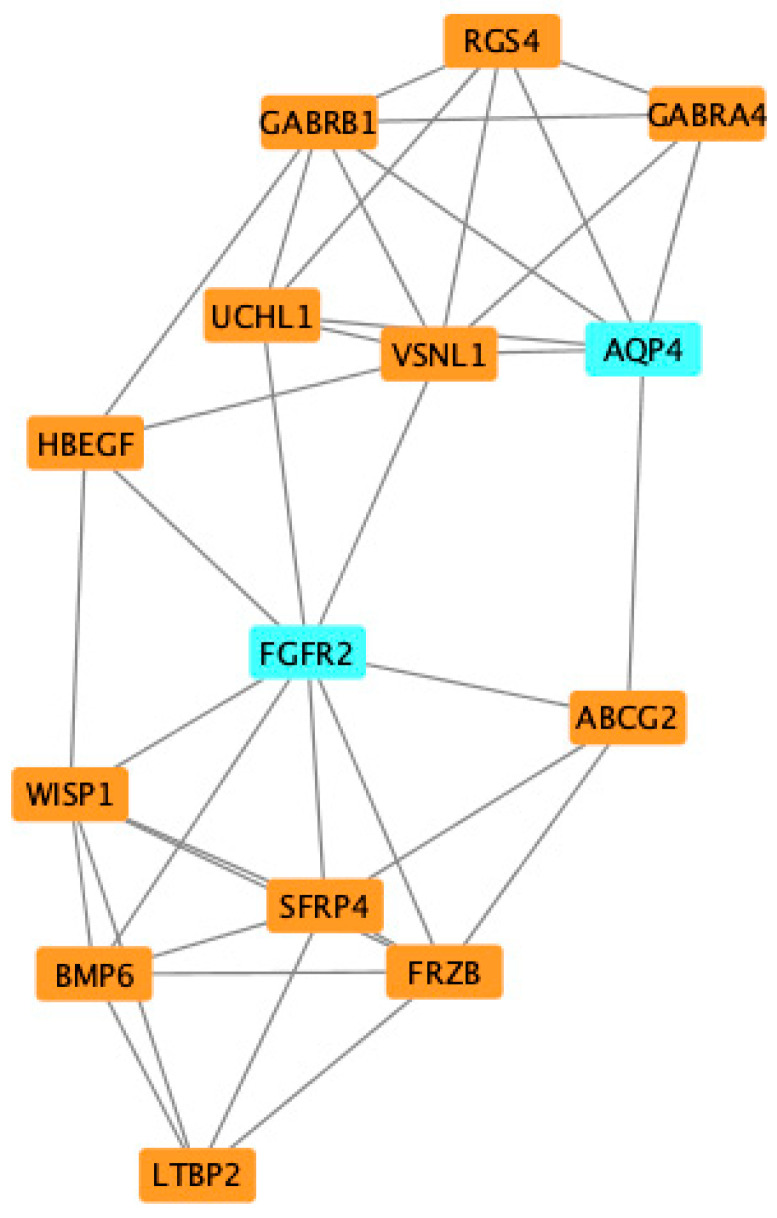
Identification of common differentially expressed hub genes (DEG) of VMC and DCM using Cytoscape and MCODE plugin.

To further explore the roles of these common DEGs, we used protein–protein interaction data from STRING to construct a network which contained 109 nodes and 306 edges (Figure 5). By importing the results of STRING into Cytoscape, we identified 14 hub genes: FGFR2, AQP4, LTBP2, BMP6, WISP1, SFRP4, FRZB, ABCG2, HBEGF, UCHL1, VSNL1, GABRB1, GABRA4, and RGS4 (Figure 6), of which FGFR2 and AQP4 were down-regulated DEGs and others were up-regulated DEGs. The proteins in the network were mainly enriched in regulation of WNT signaling pathway (GO-ID:0030111, *p* = 0.0413), cellular response to stimulus (GO-ID:0051716, *p* = 0.0262), and GABA-gated chloride ion channel activity (GO-ID:0022851, *p* = 0.0467).

### 3.4. Screening for Potential Pharmacological Targets

Potential targeted drugs of these hub genes were searched in the cMAP database, and the drugs were sorted and screened according to their connectivity score. The top 10 compounds suggested for these common DEGs were HG-6-64-01, selamectin, tetrindole, PD-198306, elvitegravir, amsacrine, GSK-461364, gefitinib, amlodipine, and AZ-628 (Table 7).

### 3.5. Subgroup Analysis

**Figure 7:**
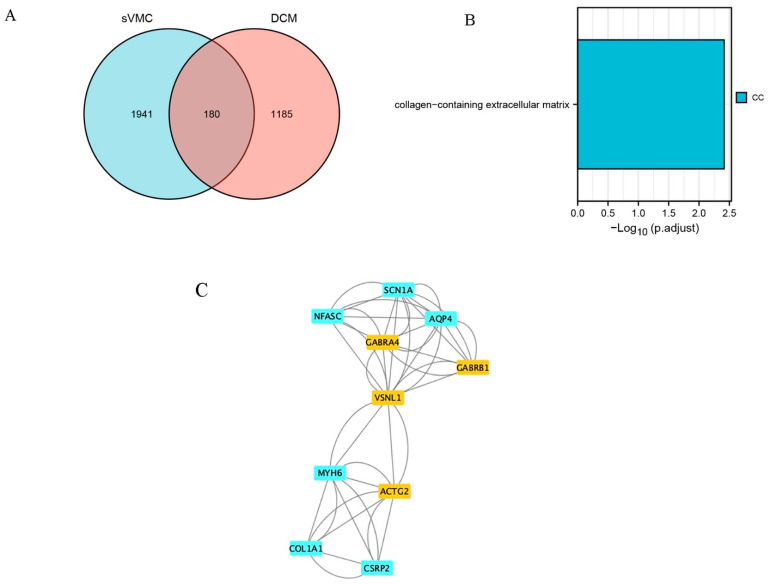
Subgroup analysis for severe myocarditis (sVMC). (**A**) Venn plot of common differentially expressed genes (DEGs) of sVMC and DCM. (**B**) Results of GO/KEGG enrichment analysis of the common DEGs of sVMC and DCM. (**C**) Identification of hub common DEGs of sVMC and DCM by using Cytoscape and MCODE plugin.

From the GSE4172 dataset, four VMC patients with EF < 40% formed the sVMC subgroup. Their differential gene analysis with four normal controls revealed a total of 2121 DEGs. There were 180 common DEGs for sVMC and DCM (Figure 7A). GO/KEGG analysis showed that these genes were mainly enriched in the collagen-containing extracellular matrix (Figure 7B). A PPI network containing 143 nodes and 447 edges were constructed. By importing these data into Cytoscape, we identified 10 hub genes (4 upregulated and 6 downregulated): VSNL1, GABRA4, GABRB1, ACTG2, SCN1A, NFASC, AQP4, MYH6, COL1A1 and CSRP2, respectively (Figure 7C). The proteins encoded by these genes were mainly clustered in the GABA-A receptor complex (GO:1902711) and myosin filaments (GO:0032982).

## 4. Discussion

Dilated cardiomyopathy is a heterogeneous group of diseases that can be divided into two categories: primary (familial inheritance) and secondary. Viral myocarditis has traditionally been considered the most common cause of secondary dilated cardiomyopathy [14,15]. However, the pathogenesis of transition from viral myocarditis to dilated cardiomyopathy is poorly understood, and the key genes and pathways among this pathophysiological process await further discovery. In this study, we first obtained the common DEGs between VMC and DCM based on gene chips from myocardial biopsy. A total of 191 DEGs of VMC and DCM were unearthed.

When compared with the studies that originally generated the reevaluated datasets, the original study that generated the GSE4172 dataset [12] found that cysteine-rich angiogenic inducer 61 (CYR61) and adiponectin (APN) played an important role in inflammatory cardiomyopathy caused by parvovirus B19-infected viral myocarditis. However, in our study, the APN gene was not among the DEGs. Although CYR61 was among the DEGs, it was not found in the final common DEGs, indicating that although CYR61 and APN may play an important role in viral myocarditis, they are not the key genes in the progression of viral myocarditis to dilated cardiomyopathy. The original study that generated the GSE4172 dataset [13] compared the differential genes of responders and non-responders to immunoadsorption with subsequent immunoglobulin G substitution (IA/IgG) treatment in 40 DCM patients with normal controls. Because our study overall compared genetic differences between DCM patients and normal controls, the results of the DEGs differed from this study.

GO enrichment analysis and KEGG pathway analysis showed that the common upregulated GEGs are mainly involved in GABA receptor complex, GABA−gated chloride ion channel activity, xenobiotic transmembrane transporter activity, and GABAergic synapse, indicating that GABA-related pathway functions are enhanced in VMC and DCM patients. This is a novel finding. Currently, it is known that γ-aminobutyric acid (GABA) is widely distributed in the cardiovascular system activity regulatory region of the central nervous system (CNS) and directly regulates cardiac sympathetic nerve activity (C-SNA) [16,17]. On the other hand, there is strong evidence that cardiac sympathetic hyperactivation is associated with DCM [18,19,20]. Therefore, whether GABA dysregulation in VMC leads to the development of DCM through this pathophysiological mechanism is worth exploring.

For the common downregulated DEGs, GO enrichment analysis found these genes were mainly enriched in ventricular cardiac muscle tissue morphogenesis and ventricular cardiac muscle tissue development, which means that ventricular myocardial tissue development are suppressed in patients with VMC and DCM. The KEGG pathway analysis found that these downregulated DEGs were mainly enriched in Rap1 signaling pathway. Rap1 (Ras-proximate-1) is a small GTPase protein which plays a crucial role in mediating cAMP signaling in isolated cardiac tissues and cell lines [21]. Similar to our study, a recent study in found a significant enrichment in Rap1 signaling pathway in VMC mice [22]. Further, basic studies show that suppression of Rap1 impairs cardiac myofibrils and conduction system in zebrafish [23]. Our study suggests that inhibition of the Rap1 signaling pathway in VMC may contribute to the progression of DCM.

Fourteen genes were identified as hub genes in the progression from VMC to DCM. Of them, FGFR2 (Fibroblast growth factor receptor 2) is a tyrosine-protein kinase that plays an essential role in the regulation of cell proliferation, differentiation, migration and apoptosis [24]. AQP4 (Aquaporin-4) forms a water-specific channel and plays an important role in brain water homeostasis and in glymphatic solute transport [25]. LTBP2 (Latent-transforming growth factor beta-binding protein 2) may play an integral structural role in elastic-fiber architectural organization and/or assembly [26]. BMP6 (Bone morphogenetic protein 6) plays an important role in bone formation and iron metabolism [27]. WISP1 (WNT1 inducible signaling pathway protein 1) is a downstream regulator in the Wnt/Frizzled-signaling pathway and is associated with cell survival [28]. SFRP4 (Secreted frizzled-related protein 4) and FRZB (frizzled related protein) are both soluble frizzled-related proteins (sFRPS) that function as modulators of Wnt signaling through direct interaction with Wnts. [29,30]. ABCG2 (ATP-binding cassette subfamily G member 2) is a broad substrate-specificATP-binding cassette transporter that plays an important role in porphyrin homeostasis and the cellular export of heme [31]. HBEGF (heparin binding EGF-like growth factor) is a Growth factor that may be involved in macrophage-mediated cellular proliferation and fibroblast mitosis [32]. UCHL1 (Ubiquitin carboxyl-terminal hydrolase isozyme L1) involved both in the processing of ubiquitin precursors and of ubiquitinated proteins. [33]. VSNL1 (Visinin-like protein 1) regulates the inhibition of rhodopsin phosphorylation in a calcium-dependent manner [34]. GABRB1 (Gamma-aminobutyric acid receptor subunit beta-1) and GABRA4 (Gamma-aminobutyric acid receptor subunit alpha-4) are both components of receptor for GABA, which functions as a ligand-gated chloride channel, and play an important role in ion transport [35,36]. RGS4 (Regulator of G-protein signaling 4) inhibits signal transduction by increasing the GTPase activity of G protein alpha subunits, which is associated with negative regulation of cardiac muscle cell development [37]. PPI network showed that these hub genes were mainly clustered in regulation of WNT signaling pathway and GABA-gated chloride ion channel activity. Consistent with our study, several studies have reported the downregulation of the Wnt pathway in animal models of DCM. In contrast, activation of the Wnt pathway in different ways helps prevent DCM development [38,39,40]. Our findings suggest that the suppression of the Wnt signaling pathway may play an important role in the progression of VMC to DCM. These hub genes may be potential therapeutic targets for the protection against progression of VMC to DCM.

As shown in Table 7, the top 10 agents predicted by cMAP for these hub genes were more concentrated in the RAF/MEK/MAPK signaling axis. To evaluate the feasibility of these drugs, HG-6-64-01 has not yet entered clinical trials. Selamectin is a potent nematocide but is highly toxic (neurotoxic). Tetrindole, as a selective inhibitor of monoamine oxidase A, is a new type of antidepressant but has a marked negative effect on detoxification. PD198306 is a selective inhibitor of MEK-1/2, which has not been used in clinical practice. Elvitegravir is a human immunodeficiency virus (HIV) integrase inhibitor which is used largely in a four-drug combination with cobicistat, emtricitabine, and tenofovir as therapy of HIV infection, but no cardiovascular applications have been reported. Az-628 is an RAF inhibitor that has not yet entered clinical trials. Amsacrine is an acridine derivative that is active in the treatment of acute leukemias and lymphomas, but no cardiovascular applications have been reported. GSK461364 is an ATP-competitive inhibitor of polo-like kinase 1 (Plk1) that currently has only one completed phase I clinical trial for non-Hodgkin’s lymphoma. Gefitinib, an EGFR inhibitor, is widely used in the treatment of non-small cell lung cancer but not cardiovascular disease. Amlodipine, as a calcium channel blocker, is one of the most widely used antihypertensive drugs. Although calcium channel blockers have not been shown to be beneficial for the treatment of patients with heart failure, several studies have shown a favorable effect of amlodipine on nonischemic dilated cardiomyopathy [41,42]. In addition, amlodipine has been reported to have a protective effect on myocardial injury in mice with congestive heart failure caused by viral myocarditis, and this therapeutic effect may be in part resulting from the inhibition of NO overproduction [42]. In conclusion, only amlodipine is currently viable among these potential therapeutic agents. Clinical trials are needed to verify its effectiveness in preventing the progression of viral myocarditis to dilated cardiomyopathy.

Because of evidence of a strong association between severe myocarditis and dilated cardiomyopathy, we created a subgroup of patients with severe myocarditis. Excitingly, the subgroup analysis yielded some novel findings. Firstly, the common DEGs of cVMC and DCM were enriched in the collagen-containing extracellular matrix, which was not found before. Currently, there is evidence that extracellular matrix remodeling plays an important role in dilated cardiomyopathy [43]. Our study suggests that viral myocarditis may develop dilated cardiomyopathy through changes in extracellular matrix related genes. Second, AQP4, VSNL1, GABRA4, and GABRB1 were still among the hub genes obtained by subgroup analysis, indicating that these genes were strongly associated with dilated cardiomyopathy caused by myocarditis. Among the newly discovered hub genes, ACTG2 was up-regulated and the rest were down-regulated. ACTG2 is involved in coding for actin, which is a major component of the sarcomere and plays an important role in cardiac contraction [44]. SCN1A encodes Sodium channel protein type 1 subunit alpha, which mediates the membrane permeability of voltage-dependent sodium channels and plays an important role in the water–sodium balance [45]. NFASC encodes Neurofascin, an ankyrin-binding protein that promotes cell adhesion [46]. Myosin-6 encoded by MYH6 is an actin-based motor protein that plays an important role in myocardial contraction [47]. Col1A1 encodes the collagen alpha-1 (1) chain, which plays an important role in extracellular matrix and fiber formation [48]. CSRP2 encodes cysteine and glycine-rich protein 2, which promotes smooth muscle cell proliferation and dedifferentiation [49]. Thirdly, the PPI network showed that these genes were clustered in the GABA-A receptor complex and myosin filaments. This is consistent with our previous findings of global hub genes clustering at GABA receptors, whereas myosin filament clustering is a novel finding. Combined with the functions of these hub genes, we can infer that severe myocarditis develops dilated cardiomyopathy through genetic changes related to sarcomere dysfunction and extracellular matrix remodeling. However, these need to be verified experimentally.

For dilated cardiomyopathy, the known and recognized mutated genes need to be taken seriously. Currently the major mutations identified in familial dilated cardiomyopathy include TIN, LMNA, MYH7, MYH6, TNNT2, ACTC1, BAG3, DSP, MYBPC3, RBM20, SCN5A, and TPM1. Our differential gene analysis did detect MYH7 and MYH6, and MYH6 was also among the hub genes of the severe myocarditis cohort. However, no other known genes were detected, probably because most of the included patients in the original study producing GSE17800 had sporadic dilated cardiomyopathy.

There are several limitations in this study. Firstly, the data set for myocarditis in this study used parvovirus B19 myocarditis samples, and whether it is representative is debatable. However, this is the largest genome-wide sample of viral myocarditis currently available, and the Venn method of screening for common DEGs has itself largely eliminated confounding factors such as different viral infections. Secondly, there were no RNAseq data, which limits the detection of moderate/weak expression changes. Unfortunately, there are currently no high-throughput datasets from myocardial biopsy samples from human viral myocarditis. The lack of RNAseq data does reduce the sensitivity of this study, but in turn increases the specificity of the detection of differential expression. Thirdly, our study lacks protein expression data and thus the most direct representation of the biological process of VMC to DCM. We will add the content of proteomics in a subsequent study. Fourthly, although we screened out potential therapeutic compounds targeting hub genes, most of them seemed infeasible, and thus further verification by animal experiments is required.

## 5. Conclusions

In conclusion, our study is the first to identify hub genes in the progression of VMC to DCM, and reveals the important role of GABA-gated chloride ion channel activity, RAP1 signaling pathway, and WNT signaling pathway. Hub genes of severe VMC and DCM include VSNL1, GABRA4, GABRB1, ACTG2, SCN1A, NFASC, AQP4, MYH6, COL1A1, and CSRP2, which mainly cluster in extracellular matrix remodeling and sarcomere dysfunction. These genes can be considered as genetic markers of early high-risk myocarditis. Amlodipine is a potential viable drug in preventing the progression of VMC to DCM. Further basic studies are needed to verify the role of these genes and pathways in the progression of VMC to DCM.

## Figures and Tables

**Table 1:** Detailed data of GSE4172 and GSE17800.

Sequence Number of Chip	GSE4172	GSE17800
Platform	GPL570	GPL570
Disease	VMC	DCM
Chip provider	Patricia Ruiz Lab of Max Planck Institute for Molecular Genetics, Germany	Funktionelle Genomforschung LAb of Universitätsmedizin Greifswald, Germany
Address	Ihnestrasse 65, Berlin	Jahnstraße 15a, Greifswald
Research object	Human	Human
Experiment type	Expression profiling by array	Expression profiling by array
Sample type	Myocardial biopsy	Myocardial biopsy
Biopsy method	EMBs from the right ventricular septum by standard procedure	EMBs in accordance with the Dallas criteria
Number of chip samples (used/total)	12/12	48/48
Number of cases/controls	8/4	40/8
Clinical diagnosis	PVB19 infected viral myocarditis	Dilated cardiomyopathy in chronic heart failure
Age (years)	52.5 ± 17	49 ± 10
Genetic DCM	-	None available
NYHA classification (n) II III	None available	21 19
LVEF (%)	42 ± 9.9	33 ± 6
Time of uploading chip	Public on 1 December 2012	Public on 31 May 2006

**Table 2:** A part of the major differentially expressed genes in GSE4172.

Gene	Log (Fold Change (FC))	*p*-Value	Adjusted *p*-Value (Adj. *p*-Value)
Ribosomal protein S4 Y-linked 1 (RPS4Y1)	4.64160144	0.045674364	0.329086230
PRKAG2 antisense RNA 1 (PRKAG2-AS1)	3.30130593	5.04 ×10^−6^	0.036385392
Long intergenic non-protein coding RNA 619 (LINC00619)	3.17781081	0.001389913	0.111583803
Lysine demethylase 5D (KDM5D)	3.07836573	0.047057584	0.331496365
Cytochrome P450 family 7 subfamily A member 1 (CYP7A1)	−2.9602019	0.009582648	0.190923364
Adiponectin, C1Q and collagen domain containing (ADIPOQ)	−2.9003638	0.005991097	0.163809624
Selectin E (SELE)	2.8820485	0.001643719	0.116984950
Carboxyl ester lipase (CEL)	−2.8475079	0.001768902	0.116984950
Odd-skipped related transcription factor 1 (OSR1)	−2.7789156	0.001293961	0.110794568
Death domain containing 1 (DTHD1)	−2.7152671	0.004448966	0.150065986

**Table 3:** A part of the major differentially expressed genes in GSE17800.

Gene	Log [Fold Change (FC)]	*p*-Value	Adjusted *p*-Value (Adj. *p*-Value)
Natriuretic peptide B (NPPB)	4.05015683	0.000115140	0.020903873
Natriuretic peptide A (NPPA)	2.75622823	0.000500400	0.033619529
Secretoglobin family 1D member 2 (SCGB1D2)	−2.57902288	6.78 × 10^−5^	0.019568598
BRAF-activated non-protein coding RNA (BANCR)	2.41882011	0.010147490	0.144758837
SH3 domain containing GRB2 like 2 (SH3GL2)	2.35313533	3.26 ×10^−5^	0.015148092
Dickkopf WNT signaling pathway inhibitor 2 (DKK2)	2.19390935	0.001508777	0.057371767
Tryptase alpha/beta 1 (TPSAB1)	2.16841600	2.59 × 10^−7^	0.001867599
Tubulin tyrosine ligase like 2 (TTLL2)	−2.08152092	0.000291571	0.027683044
Transmembrane protein with EGF like and two follistatin like domains 2 (TMEFF2)	−2.06265730	0.003409397	0.08621005
Ankyrin repeat domain 33B (ANKRD33B)	2.04754302	1.72 × 10^−5^	0.010465682

**Table 4:** A part of the common differentially expressed genes of VMC and DCM.

Gene	GSE4172			GSE17800		
	LogFC	*p*-Value	Adj. *p*-Value	LogFC	*p*-Value	Adj. *p*-Value
Secretoglobin family 1D member 2S (SCGB1D2)	−1.75498617	0.04204305	0.31896103	−2.57902288	6.78 × 10^−5^	0.01956859
Tubulin tyrosine ligase like 2 (TTLL2)	−1.82673252	0.03890554	0.30862780	−2.08152092	0.00029157	0.02768304
Carbohydrate sulfotransferase 9 (CHST9)	−2.19566055	0.01626551	0.22897841	−1.63510255	0.00283399	0.07848970
Ankyrin repeat domain 33B (ANKRD33B)	1.68900071	0.00959245	0.19092336	2.04754302	1.72 × 10^−5^	0.01046568
Attractin like 1 (ATRNL1)	1.91097325	0.00197345	0.11875941	1.72884700	9.96 × 10^−7^	0.00359621
leucine rich repeat transmembrane neuronal 4 (LRRTM4)	−2.39878360	0.02226852	0.25514298	−1.22008170	0.00191667	0.06546316
LOC101927256	−2.29163892	0.00192950	0.11875941	−1.30036608	0.00737064	0.12564681
C3orf67	−2.01670334	0.01670482	0.23158961	−1.56047472	0.00573556	0.11059997
Sphingosine-1-phosphate phosphatase 2 (SGPP2)	−2.45705659	0.03309688	0.29296064	−0.97853812	0.04971122	0.29650900
AC124997.1	−1.49892026	0.04662649	0.33039811	−1.88192387	0.00051151	0.03366817

VMC, viral myocarditis; DCM, dilated cardiomyopathy.

**Table 5:** GO enrichment and KEGG pathway analysis for the common up-regulated differentially expressed genes of VMC and DCM.

Category	ID	Term	Count	*p*-Value
BP	GO:1903792	negative regulation of anion transport	3/48	4.99 × 10^−5^
	GO:0042220	response to cocaine	3/48	3.81 × 10^−4^
	GO:0060359	response to ammonium ion	4/48	3.98 × 10^−4^
	GO:0051956	negative regulation of amino acid transport	2/48	4.20 × 10^−4^
	GO:0060078	regulation of postsynaptic membrane potential	4/48	4.56 × 10^−4^
CC	GO:1902711	GABA-A receptor complex	2/51	0.001
	GO:1902710	GABA receptor complex	2/51	0.001
	GO:0097730	non-motile cilium	3/51	0.007
	GO:0034707	chloride channel complex	2/51	0.007
	GO:0034702	ion channel complex	4/51	0.008
MF	GO:0022851	GABA-gated chloride ion channel activity	2/48	5.51 × 10^−4^
	GO:0042910	xenobiotic transmembrane transporter activity	2/48	5.51 × 10^−4^
	GO:0004890	GABA-A receptor activity	2/48	0.001
	GO:0099095	ligand-gated anion channel activity	2/48	0.001
	GO:0016917	GABA receptor activity	2/48	0.002
KEGG	hsa04727	GABAergic synapse	3/25	0.002
	hsa05032	Morphine addiction	3/25	0.003
	hsa05033	Nicotine addiction	2/25	0.007
	hsa05219	Bladder cancer	2/25	0.007

**Table 6:** GO enrichment and KEGG pathway analysis for the common down-regulated differentially expressed genes of VMC and DCM.

Category	ID	Term	Count	*p*-Value
BP	GO:0055010	ventricular cardiac muscle tissue morphogenesis	3/44	1.96 × 10^−4^
	GO:0003229	ventricular cardiac muscle tissue development	3/44	2.94 × 10^−4^
	GO:0055008	cardiac muscle tissue morphogenesis	3/44	5.74 × 10^−4^
	GO:0003208	cardiac ventricle morphogenesis	3/44	6.77 × 10^−4^
	GO:0048639	positive regulation of developmental growth	4/44	9.11 × 10^−4^
MF	GO:0004714	transmembrane receptor protein tyrosine kinase activity	2/44	0.010
	GO:0016829	lyase activity	3/44	0.011
	GO:0005518	collagen binding	2/44	0.012
	GO:0019199	transmembrane receptor protein kinase activity	2/44	0.016
	GO:0038024	cargo receptor activity	2/44	0.019
KEGG	hsa04015	Rap1 signaling pathway	4/23	0.003

**Table 7:** Top 10 prediction results from cMAP for the prevention against progression of VMC to DCM.

Score	Name	Description	Comment
95.81	HG-6-64-01	RAF inhibitor, Abl kinase inhibitor, ephrin receptor inhibitor, KIT inhibitor, MAP kinase inhibitor, MEK inhibitor, p38 MAPK inhibitor, src inhibitor	uncharacterized
94.20	selamectin	nematocide	neurotoxicity
92.97	tetrindole	monoamine oxidase inhibitor	antidepressant
92.63	PD-198306	MAP kinase inhibitor, MEK inhibitor	uncharacterized
91.78	elvitegravir	HIV integrase inhibitor, HIV inhibitor	classic cocktail drug ingredients
91.60	amsacrine	topoisomerase inhibitor, DNA intercalating drug	in clinical trial for Acute Myeloid Leukemia
91.53	GSK-461364	PLK inhibitor	in clinical trial for Non-Hodgkin Lymphoma
91.45	gefitinib	EGFR inhibitor	first-generation targeted drug for non-small cell lung cancer
91.13	amlodipine	breast cancer resistance protein inhibitor, calcium channel blocker, calcium channel inhibitor, L-type calcium channel blocker	classic CCB class antihypertensive drug
90.41	AZ-628	RAF inhibitor	uncharacterized

## Data Availability

GSE4172: accessed on 5 June 2022, https://www.ncbi.nlm.nih.gov/geo/query/acc.cgi?acc=GSE4172. GSE17800: accessed on 5 June 2022, https://www.ncbi.nlm.nih.gov/geo/query/acc.cgi.
